# A Comparison Between a Resuscitation Glove and Standard Manual Compressions on the Quality of Cardiovascular Resuscitation: Manikin-Based Randomized Crossover Trial

**DOI:** 10.1097/JCN.0000000000001206

**Published:** 2025-04-03

**Authors:** Desale Tewelde Kahsay, Miretta Tommila, Laura-Maria Peltonen, Eliisa Löyttyniemi, Yu Xiao, Henry Mauranen, Sanna Salanterä

**Affiliations:** 1 Desale Tewelde Kahsay, MSc, Department of Anaesthesiology and Intensive Care, Faculty of Medicine, University of Turku, Turku, Finland.; 2 Miretta Tommila, MD, PhD, Department of Perioperative Services, Intensive Care Medicine and Pain Management, Turku University Hospital and University of Turku, Turku, Finland.; 3 Laura-Maria Peltonen, PhD, Department of Nursing Science, Faculty of Medicine, Turku University Hospital and University of Turku, Turku, Finland; Department of Health and Social Management, University of Eastern Finland and Wellbeing Services County of North Savo, Kuopio, Finland.; 4 Eliisa Löyttyniemi, MSc, Department of Biostatistics, University of Turku and Turku University Hospital, Turku, Finland.; 5 Yu Xiao, DSc, Department of Information and Communications Engineering, Aalto University Espoo, Finland.; 6 Henry Mauranen, MSc, Department of Information and Communications Engineering, Aalto University Espoo, Finland.; 7 Sanna Salanterä, PhD, Professor, Department of Nursing Science, Faculty of Medicine, University of Turku and Turku University Hospital, Turku, Finland.

**Keywords:** cardiopulmonary resuscitation, cardiac arrest, feedback, medical device

## Abstract

**Background:**

Several audiovisual feedback (AVF) devices have been developed to monitor chest compression quality during cardiopulmonary resuscitation (CPR). However, most marketed stand-alone AVF devices are inflexible and rigid, causing discomfort and sometimes pain to the rescuers' hands.

**Objective:**

The objective of this study was to evaluate the effectiveness and usability of a newly developed soft and flexible resuscitation glove designed to improve the quality of chest compressions during CPR.

**Methods:**

We conducted a manikin-based randomized crossover study to compare the effectiveness of a newly developed AVF device (ResuGlove CPR Group) and standard CPR (Standard CPR Group) in improving the quality of chest compressions in simulated cardiac arrest cases. The usability of the newly developed ResuGlove was assessed using a System Usability Scale questionnaire.

**Results:**

There were no significant differences in compression depth (mean, 53.69 vs 53.28; *P* = .70) and compression rate (mean, 111.48 vs 113.38; *P* = .23) between the ResuGlove CPR and Standard CPR groups. However, the group using ResuGlove had a higher percentage of complete chest releases between compressions (*P* = .008). Furthermore, the ResuGlove CPR Group had a significantly higher percentage of participants who performed chest compressions with adequate compression depth (82.8% vs 41.4%, *P* = .001) and compression rate (96.6% vs 72.4%, *P* = .012) compared with the Standard CPR Group. The ResuGlove usability score was calculated to be 70.4.

**Conclusions:**

The newly developed ResuGlove significantly improved the quality of certain chest compression parameters, and the device’s usability score was within the acceptable range.

**What’s New and Important**

- The resuscitation glove is a textile-based soft and flexible, wearable real-time feedback device newly developed for improving chest compression quality provided by laypersons and inexperienced healthcare professionals.
- In this study, ResuGlove enhances the quality of chest recoil during simulated manikin-based cardiopulmonary resuscitation, rendering the new devices superiority to many of the previously developed standalone audiovisual feedback devices.
- This textile-based portable wearable real-time feedback device was comfortable and easy to use during the simulated manikin-based cardiopulmonary resuscitation.

Cardiac arrest is a serious medical emergency in which the heart suddenly stops beating.^1^ High-quality cardiopulmonary resuscitation (CPR) is essential during cardiac arrest to maintain the blood flow and oxygen supply to vital organs.^1^ Therefore, laypersons' immediate initiation of CPR can help save lives by sustaining artificial blood circulation until medical assistance arrives.^2^ Patients who receive timely CPR from laypersons before the arrival of emergency management teams tend to have better survival rates at hospital discharge.^3^ As a result, the 2021 European Resuscitation Council guideline recommends that every citizen be adequately trained to provide quality CPR to save lives.^4^

In this study, “laypersons” or “lay responders” refers to individuals who are not licensed healthcare professionals or certified CPR responders but may have received some training in CPR techniques.^5^ These individuals might have obtained their training through community programs or first aid courses, enabling them to provide immediate assistance until professional medical help arrives.

Many laypersons hesitate to start CPR because of a lack of training or fear of causing harm to patients.^6,7^ In a previous study,^7^ layperson bystanders were asked about their comfort level with performing CPR and their specific concerns regarding taking action to begin CPR. The primary concerns were the potential for causing injury to patients and a lack of appropriate skills. Even if they decide to start CPR, they may not know the correct technique to perform high-quality chest compressions.^8^ Benkerrou et al^9^ conducted a study to investigate whether the type of bystander administering CPR during out-of-hospital cardiac arrests was associated with patient survival after cardiac arrest. The study found that CPR performed by medically trained bystanders was associated with a higher rate of CPR initiation and a greater likelihood of survival to hospital discharge compared with CPR performed by lay bystanders.^9^

Various real-time audiovisual feedback (AVF) devices have been designed and introduced into the market to minimize chest compression errors and increase the confidence of inexperienced rescuers.^10^ These devices monitor critical chest compression parameters, including compression depth, rate, chest recoil, and hand position and give immediate AVF feedback to ensure high-quality CPR.^11^ Audiovisual feedback devices can empower laypersons and inexperienced healthcare professionals to enhance their confidence, resulting in higher-quality chest compressions that improve patient outcomes during cardiac arrest.^11,12^

Audiovisual feedback devices can be broadly classified into integrated and standalone systems.^10,11^ Integrated AVF devices are built into larger multifunctional devices, such as defibrillators, and are commonly used by qualified healthcare professionals in healthcare settings and educational institutions.^10,11^ These devices are relatively expensive, complex, and inaccessible to laypeople and healthcare professionals at the community level.^10,11^ Standalone AVF devices are portable and easy-to-use self-contained units suitable for laypeople and healthcare professionals. They are valuable for public access in developing countries and resource-limited settings.^8,10,11,13^

Most of the marketed standalone AVF devices, such as the CPR-plus,^14^ CPREzy,^15^ and CPRmeter,^16^ are inflexible and rigid in structure. They are designed to be placed between the chest and the rescuer’s hands or held manually during CPR.^14–17^ Consequently, they can potentially cause pain and discomfort to the rescuers' hands. For example, Perkins et al^15^ conducted a study to evaluate the effectiveness of the CPREzy device on the quality of chest compressions. Although the study reported improved chest compression quality with the CPREzy device, most participants (95%) experienced discomfort in the heels of their hands and wrists. In addition, 1 participant who used the CPREzy standalone device suffered a soft tissue injury. In a similar study, 8 participants (20%) reported wrist and hand discomfort while using the CPR-plus feedback device, particularly toward the end of chest compressions.^14^ Furthermore, researchers reported sustained hand injuries during CPR training when using the CPRmeter as a feedback device.^18^ In their letter to the editor, they highlighted that many trainees experienced pain and soreness in the heels of their hands, with 38.9% sustaining injuries. In addition, research by Park^17^ revealed that 48.5% of rescuers reported pain in the backs of their hands when using a handheld device (smartphone) for real-time feedback during CPR.

Smartwatch-based wearable feedback devices placed on the wrist of the rescuer have been developed as an alternative to the rigid AVF devices placed between the patient’s chest and the rescuer’s hands.^19^ Although these wearable devices have the potential to alleviate discomfort experienced by patients and rescuers, their accuracy in measuring some of the compression parameters, such as the chest recoil, gets compromised.^20^ This is because these devices are placed on the rescuer’s wrist or arm, far from where the compression is applied.^21,22^

Researchers from the University of Turku and Aalto University have developed flexible, soft, and portable resuscitation gloves (ie, the ResuGlove) that are compatible with the human body to address the aforementioned limitations.^23^ The ResuGlove has 3 pressure sensors on the palm and an inertial measurement unit on the dorsal side, allowing closer contact with the compression site during CPR.^23^ In this study, our aim was to evaluate the effectiveness and usability of the newly developed ResuGlove in improving the quality of chest compressions during simulated cardiac arrest on training manikins.

## Methods

### Design

We conducted a prospective randomized crossover study to compare the effectiveness of ResuGlove with Standard CPR in improving the quality of chest compression parameters during simulated cardiac arrest cases. A crossover design is more efficient than a parallel design, as it requires fewer participants to achieve the same level of statistical power by allowing individuals to serve as their own controls.^24^ Furthermore, between-subject variability can be minimized in group comparisons, and the allocation imbalances often observed in parallel randomized controlled trials are rarely present in randomized crossover designs.^24^ Moreover, the typical disadvantages associated with crossover designs, such as high dropout rates after enrollment and possible carryover effects,^24^ were not anticipated in this study. To assess usability, participants completed the System Usability Scale questionnaire immediately after delivering 2 minutes of CPR using the newly developed ResuGlove. The System Usability Scale questionnaire is a reliable and widely recognized tool for assessing the usability of various technologies and innovations (Supplemental Digital Content, Supplementary Data 1, http://links.lww.com/JCN/A344).^25–28^

The research received approval from the Ethics Committee for Human Sciences at the University of Turku. The researchers then obtained permission from Turku University of Applied Sciences to collect data. Researchers assured participants' autonomy by allowing them to voluntarily determine their participation, including the right to withdraw without explaining themselves. Participants received no direct benefits from taking part in the study. No significant risks were anticipated other than potential discomfort caused by the tightness of the ResGlove during chest compressions. On the data collection day, each participant received a thorough oral explanation of the study. Then, all participants signed the paper-based informed consent form prior to engaging in the study.

### Participants and Recruitment

Researchers recruited volunteer nursing students from Turku University of Applied Sciences. All bachelor nursing students who had completed basic life support training were eligible to participate in the study. Our study is in early development; therefore, we decided to include bachelor’s nursing students with basic CPR training as our test group. This provides a more controlled environment and allows students to offer valuable feedback for further development of the devices. In addition, in out-of-hospital cardiac arrest situations, bystanders with basic training in CPR are more likely to attempt resuscitation.^5^ Hence, including nursing students who have received basic CPR training guarantees that the outcomes are relevant to real-world contexts, where CPR feedback devices can offer the greatest advantage. Students with health issues affecting performance, such as cardiac or pulmonary diseases, back pain, or wrist problems, were excluded from the study. The researchers contacted nursing students by sending an invitation letter to their institutional email addresses. The letter provided information about the purpose of the study, confidentiality, inclusion and exclusion criteria, and trial design. Students interested in participating were asked to respond via email or a short message service using the contact information in the invitation letter. The study started in May 2023 and ended in September 2023.

### Sample Size and Randomization

The researchers conducted a randomized crossover pilot study with 8 participants in autumn 2022, and the sample size was calculated based on the information from our unpublished pilot study. The sample size for the crossover randomized trial was determined based on the mean chest compression depth from the pilot study. It was found that a minimum mean difference of 3.5 mm could be detected with a standard deviation (SD) of 5.7 (within-subject SD) at a significant level of 0.05 (2-sided) and a power of 0.8. Considering an estimated dropout rate of 20%, the total sample size was set to 30. One participant withdrew from the study without providing a reason. Consequently, 29 participants were included in the study and randomly assigned to the group sequence.

**FIGURE 1.**
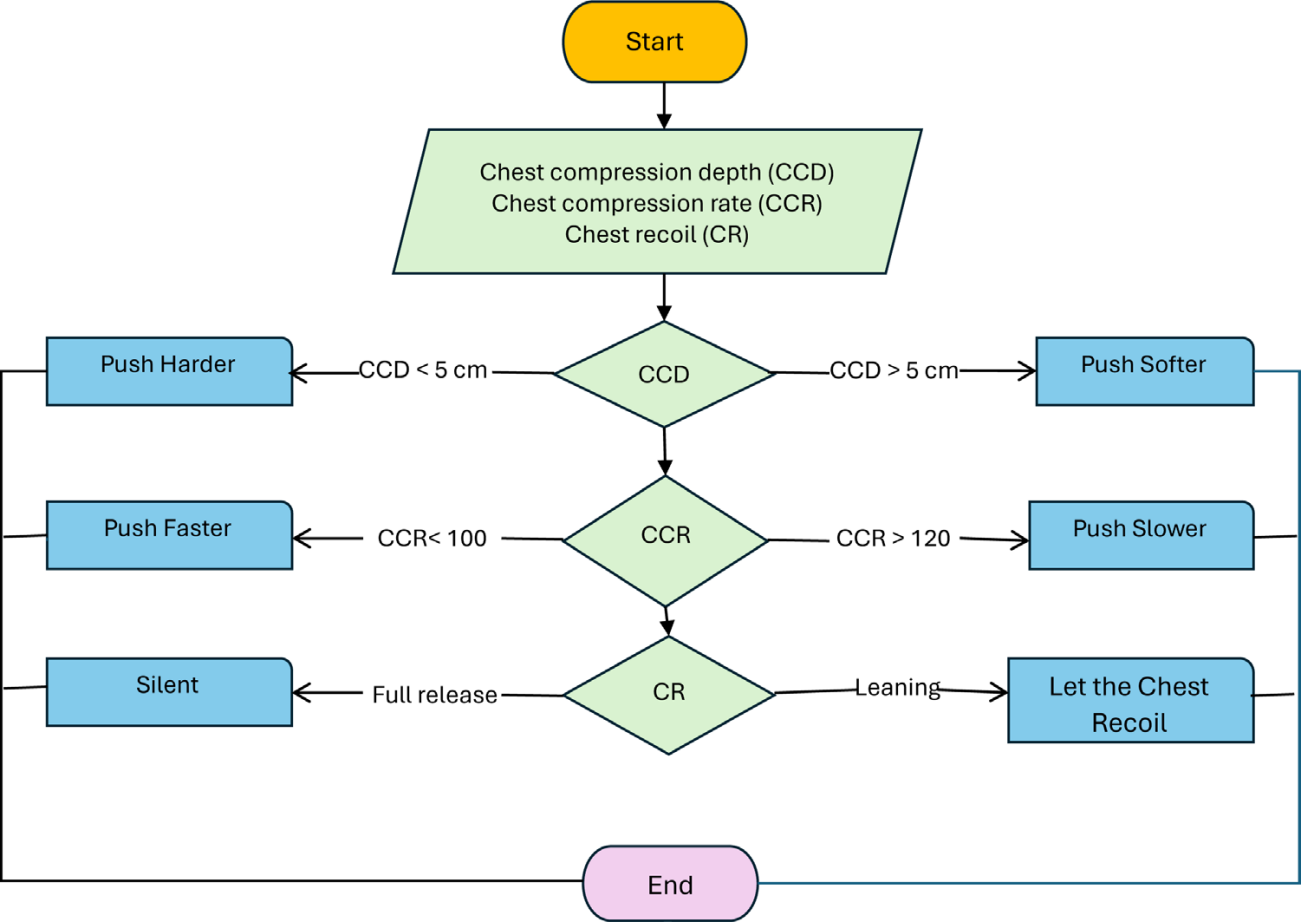
Flowchart of the ResuGlove audio feedback. A laptop connected to the ResuGlove provided real-time audio feedback for compression depth, rate, and chest recoil.

An online-based random number generator software (https://www.randomizer.org/) randomly allocated participants into 2 group sequences, ensuring a fair and unbiased process. This approach guaranteed that 14 participants started with ResuGlove CPR and the other 15 with standard CPR.^29^ The principal investigator randomly assigned participants to different sequences (details are described in the intervention). After a 30-minute recovery period, participants switched to the other group.

### Research Materials and Setting

Data were collected at the Health Campus University of Turku Simulation Center, which is shared by the Turku University of Applied Sciences, the University of Turku, and the Turku University Hospital.

The CPR training manikin (Resusci Anne Quality Cardiopulmonary Resuscitation (QCPR), Laerdal Medical) was used to perform the CPR. The manikin was placed on a hard floor surface when participants performed compressions while kneeling close to the manikin. The 2-minute CPR of both groups was recorded using the Laerdal QCPR feedback system. The Laerdal QCPR feedback system is an app-based real-time visual feedback system that can be wirelessly connected to a training manikin (Laerdal Resusci Anne) and provides feedback on the quality of chest compressions, including chest compression depth, chest compression rate, and chest recoil. More information about the Resusci Anne QCPR manikin and the Laerdal QCPR feedback system can be retrieved from the manufacturer’s website.^30^ The data were extracted from the QCPR app and transferred to Microsoft Excel for cleaning to fix possible errors and formatting inconsistencies before being moved to the Statistical Analysis System (SAS) software for analysis. The transferred data were meticulously reviewed for accuracy to ensure that all values fell within the expected range, specifically, a compression depth between 50 and 60 mm and a rate between 100 and 120 per minute. Any extreme values indicating typographical errors or inconsistencies in units (such as mm versus cm) were thoroughly examined and corrected before transferring the data to SAS software for analysis. The initial data cleaning was conducted by the first author and subsequently verified by a second author.

The newly developed ResuGlove was used as a feedback device to guide rescuers in the experiment group (ResuGlove CPR Group).^23^ The ResuGlove CPR Group received audio commands from the ResuGlove at the beginning (start the compression) and at the end (session is finished) of the 2-minute compression, along with feedback on chest compression performance.

The tested prototype of the ResuGlove was developed in collaboration between the University of Turku and Aalto University.^23^ It is designed to be worn on the rescuer’s hands and provide audio feedback on chest compression quality, compression depth, compression rate, chest recoil, and interruptions during CPR. This lightweight, soft, and hand-adaptable ResuGlove overcomes the limitations of other rigid, freestanding AVF feedback devices.^23^

The newly developed ResuGlove was used as a feedback device to guide rescuers in the experiment group (ResuGlove CPR Group).^23^ Three pressure sensors were integrated into the palm of the right-hand glove, positioned to face the chest during CPR. These pressure sensors were complemented with an inertia measurement unit that captures motion and orientation using an accelerometer to enhance the accuracy of detecting compression depth and rate. The inertia measurement unit was mounted to the glove on the left hand. All sensors were linked to a single Arduino UNO, which was then connected to a laptop to generate real-time audio feedback.^23^

ResuGlove connected to the computer provided audio corrective feedback when the chest compressions were not performed according to the 2021 European Resuscitation Council guidelines. For compression depth, the feedback was to push harder if the compression depth was less than 50 mm and softer if it was deeper than 60 mm (Figure 1). For compression rate, the feedback was to push faster if the rate was less than 100 per minute and slower if it was greater than 120 per minute (Figure 1). The new device also provided audio feedback to “let the chest recoil” if the chest was not fully released between each compression.

**FIGURE 2.**
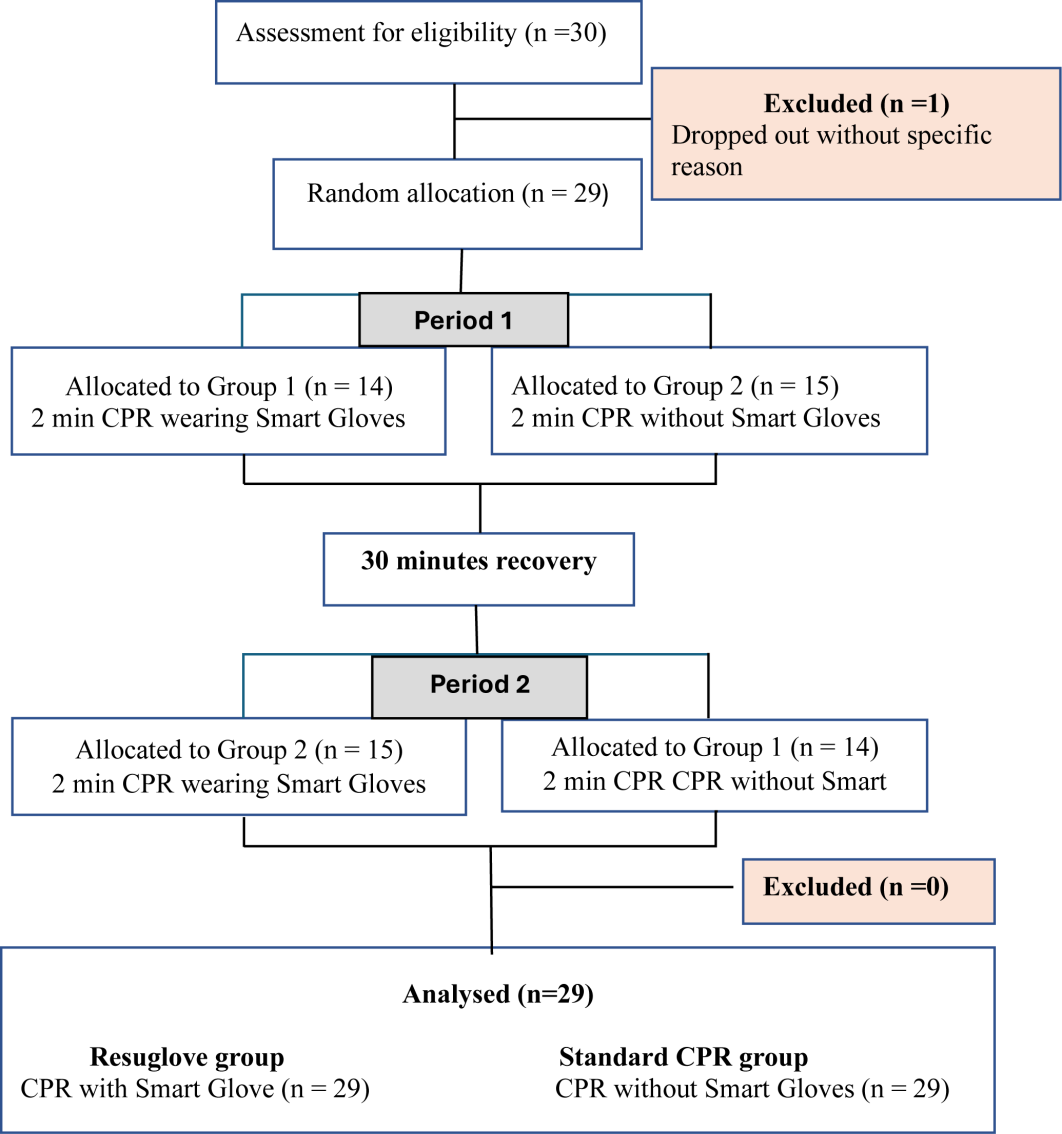
Flow diagram for crossover trials. Period 1: Group 1 performed 2 minutes of CPR wearing ResuGlove, and group 2 performed 2 minutes of chest compression without ResuGlove. Period 2: Group 1 performed 2 minutes of chest compressions without ResuGlove, and group 2 performed 2 minutes of CPR wearing ResuGlove.

### Intervention

This was a crossover randomized mankind-based trial, a design chosen to ensure that all selected participants received both interventions, thereby reducing the potential for bias. Before performing CPR, a professional with extensive experience and expertise showed all participants briefly how to perform CPR using the ResuGlove prototype and how to respond to the feedback from the device. Then, participants were randomly allocated into groups 1 and 2 using the random generator software described previously. In period 1, group 1 was assigned to perform continuous hands-on CPR for 2 minutes wearing the ResuGlove with an ongoing feedback program, whereas group 2 performed the same task as group 1 but without the ResuGlove. In period 2 (after a 30-minute recovery period), group 1 performed continuous hands-on CPR for 2 minutes without the ResuGlove, whereas group 2 performed continuous hands-on CPR for 2 minutes wearing the ResuGlove with an ongoing feedback program. During the 2-minute CPR, only 1 participant was observed at a time, and the researcher did not intervene in any of the cases. Finally, participants who performed CPR while wearing the ResuGlove (period 1, group 1; period 2, group 2) with real-time feedback program were analyzed as the ResuGlove CPR Group. Those who performed CPR without the ResuGlove (period 1, group 2, and then period 2, group 1) were analyzed as the Standard CPR Group (Figure 2). The Consolidated Standards of Reporting Trials (CONSORT) flow diagram (Figure 2 illustrates the recruitment process, randomization, sequence of allocation to the 2 groups, and the number of participants included in the final analysis. The CONSORT checklist of essential items for reporting randomized crossover trials can be found in the supplementary Data (Supplemental Digital Content, Supplementary Data 2, http://links.lww.com/JCN/A345).

### Survey on the Usability of the ResuGlove

To assess usability, participants completed a paper-based System Usability Scale questionnaire immediately after delivering 2 minutes of CPR with the newly developed ResuGlove. Brooke^31^ initially developed System Usability Scale to evaluate product usability, which was later modified for various technological products.^25–28^ The System Usability Scale questionnaire is a reliable and widely recognized tool for assessing the usability of various technologies and innovations.^25–28^ It is quick and easy to administer and provides a single score on a scale that most people can understand. The System Usability Scale overall score ranges from 0 to 100, with higher scores indicating better usability. However, it is essential to remember that a System Usability Scale score is not a percentage; therefore, a score of 50 does not mean the product is “half as good” as one scoring 100.^25^ Instead, it often suggests a significant usability issue with the product at that score. The System Usability Scale questionnaire consists of 10 statements, with odd numbers phrased positively and even negatively. Respondents rate their agreement from 1 (strongly disagree) to 5 (strongly agree). To calculate the System Usability score:

- We subtracted 1 from the score of positively worded items (score − 1).
- We subtracted the score of the negatively worded items from 5 (5 − score).
- To calculate the final System Usability score, we multiplied the obtained score by 2.5 as guided by the System Usability Scale score criteria.

According to Bangor’s System Usability Scale criteria (Bangor et al^25^), System Usability scores can be interpreted in various ways. According to the acceptability category, the System Usability Scale values are divided into 3 groups: unacceptable (<50), marginal (50–70), and acceptable (>70).^25^ The ResuGlove’s usability was interpreted based on the System Usability score acceptability category provided by Bangor et al.^25^

### Measurements

The primary outcomes were the quality of the average depth, rate, and chest recoil during a 2-minute CPR session. In addition, the study evaluated the percentage of compressions with adequate depth (50–60 mm) and the participants' experience regarding the usability of ResuGlove. The chest compressions were assessed according to the European Resuscitation Council Guideline 2021 recommendations,^32^ and usability was evaluated using the System Usability Scale questionnaire.

### Data Analysis

The data were analyzed using SAS software, version 9.4 of the SAS System for Windows (SAS Institute Inc, Cary, NC). Normally distributed data were reported as mean and SD. Nonnormally distributed data were presented as median and interquartile range (IQR). Normality was assessed visually, together with the QQ-plot and the Shapiro-Wilk test. Linear models designed for crossover analysis, including period, carry-over, and treatment effect testing were used to analyze the data. We also assessed the period and carry-over effects for all variables that did not meet the assumptions for parametric testing. No carry-over or period effect was observed (in all cases, *P* values >.298). The Wilcoxon signed-rank test was used to analyze variables where studentized residuals did not follow a normal distribution. McNemar’s test was used to compare the percentage of participants (paired nominal data) who performed chest compressions within the recommended range. Our data analysis' significance threshold was set at a *P* value of <0.05 (2-tailed).

## Results

In the study, 29 nursing students were selected for participation, consisting of 23 women (79.3%) and 6 men (20.7%). All participants had received basic life support CPR training, with the majority 19 (65.5%) having no experience of AVF device use (Table 1).

**TABLE 1.** Participants' Demographic Characteristics

Participants	n (%)
Sex	
Women	23 (79.3)
Men	6 (20.7)
Basic life support training	
Yes	29 (100)
No	0 (0)
AVF device experience	
No	19 (65.5)
Yes	10 (34.5)
Actual CPR experience	
No	23 (79.3)
Yes	6 (20.7)
Year of study	
First year	16 (55.2)
Third year	13 (44.8)

**FIGURE 3.**
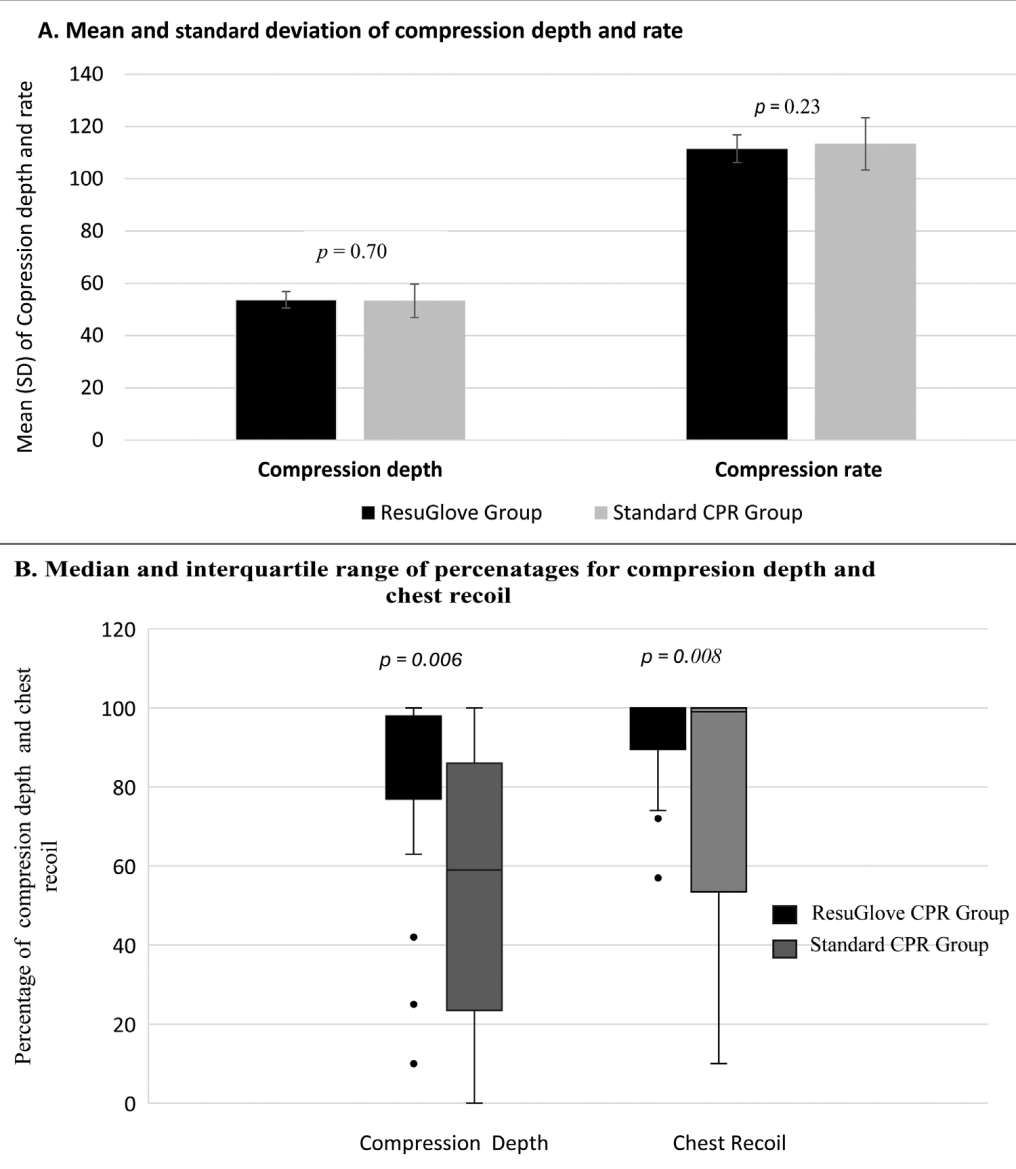
The outcomes of chest compression parameters. Figure 3A illustrates the mean and SD of mean compression depth and mean compression rate. Figure 3B illustrates the median and IQR for the percentage of compression with adequate CD and complete CR. CD, compression depth; CR, chest recoil.

No significant differences were found between the ResuGlove CPR and Standard CPR Groups in terms of mean compression depth in millimeters (ResuGlove CPR vs Standard CPR Groups, 53.69 vs 53.28; *P* = .70) and mean compression rate per minute (ResuGlove CPR vs Standard CPR Groups, 111.48 vs 113.38; *P* = .23) (Figure 3A).

**FIGURE 4.**
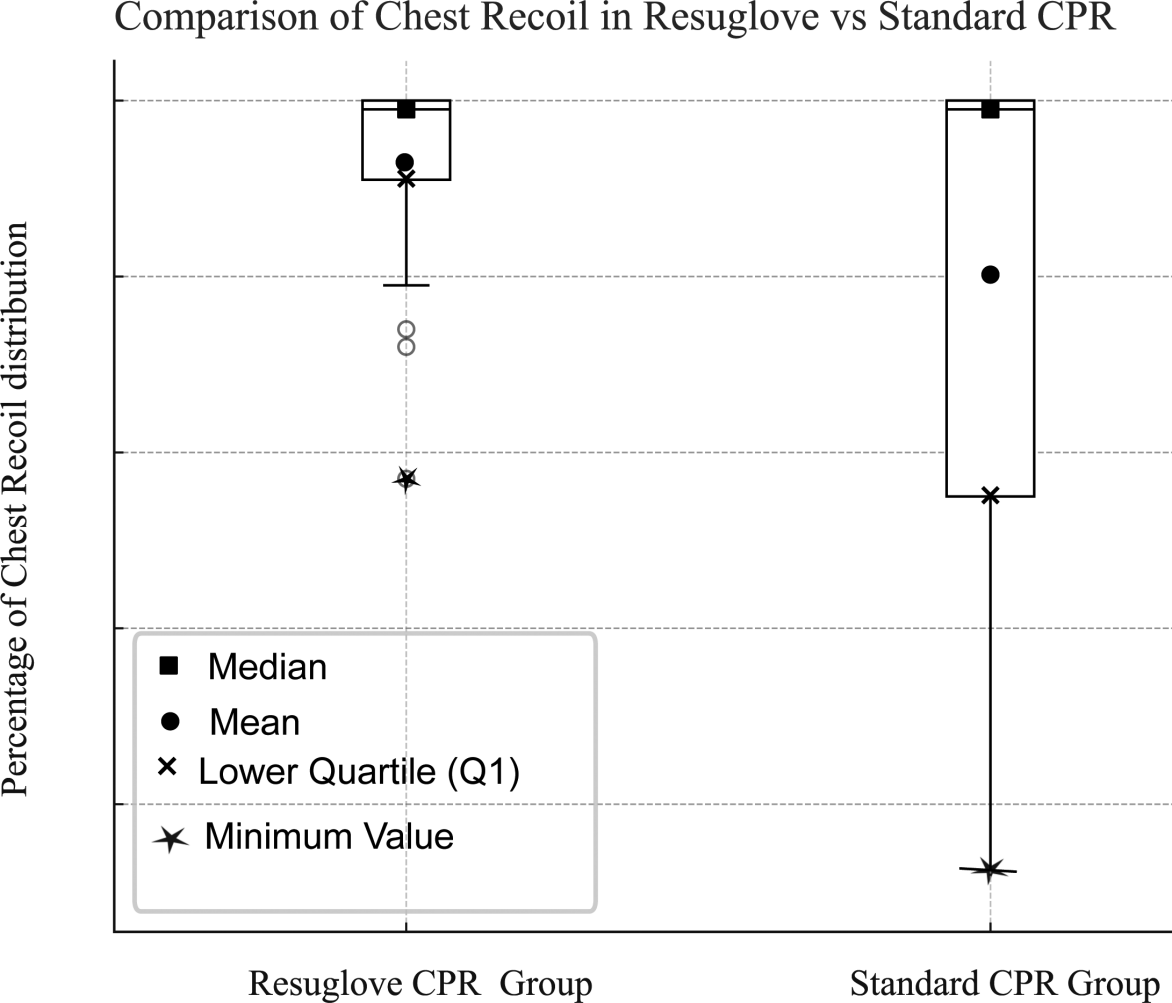
Data distributions reflect the percentage of chest recoil. The figure indicates that the median for the 2 groups is the same, but the mean and lower quartile differ in the 2 groups.

We examined the data to determine if there was a significant difference between the 2 groups regarding the percentage of compressions with adequate depth (50–60 mm) and complete chest recoil. This refers to the percentage of chest compressions that achieved adequate compression depth and full chest release. We used nonparametric tests (Wilcoxon signed-rank test) because the data for the percentage of adequate compression depth and complete chest recoil did not fulfill the assumptions of the normal data distribution. In the ResuGlove CPR group, the percentage of compressions with adequate depth was 87% (IQR, 77%–98%), compared with 59% (IQR, 23.4%–86%) in the standard CPR group, which was statistically significant (*P* = .006) (Figure 3B). In addition, the percentage of chest compressions with complete chest release was 99% (IQR, 89.5%–100%) in the ResuGlove CPR group compared with 99% (IQR, 53.5%–100%) in the Standard CPR group (*P* = .008) (Figure 3B). The median remains the same in the case of complete chest recoil. However, the finding still indicates a statistically significant difference favoring the ResuGlove CPR group (see Discussion and Figure 4 for more information).

We also analyzed the data to determine if there was a significant difference between the 2 groups in the number (percentage) of participants who performed mean chest compressions within the recommended range (Table 2). The percentage of participants achieving an adequate mean compression depth (50–60 mm) significantly improved when ResuGlove was used during CPR (ResuGlove CPR Group vs Standard CPR Group, 82.8% vs 41.4%; *P* = .001). Similarly, using ResuGloves significantly increased the percentage of participants meeting the recommended mean compression rate of 100 to 120 per min (ResuGlove CPR Group vs Standard CPR Group, 96.6% vs 72.4%; *P* = .012). No significant difference was observed between the groups in the percentage of participants who achieved complete chest recoil (ResuGlove CPR Group vs Standard CPR Group, 48.3% vs 48.3%; *P* = 1.00).

**TABLE 2.** Number (Percentage) of Participants Who Performed Adequate Chest Compressions

Outcome	ResuGlove CPR Group	Standard CPR Group	*P*
Mean compression depth, mm			0.001
Within range	24 (82.8)	12 (41.4)	
Out of range	5 (17.2)	17 (58.6)	
Mean compression rate, per min			0.012
Within range	28 (96.6)	21 (72.4)	
Out of range	1 (3.5)	8 (27.6)	
Median percentage of chest recoil			1.000
Complete release	14 (48.3)	14 (48.3)	
Incomplete release	15 (51.7)	15 (51.7)	

In the table, the term “within range” refers to when the mean compression depth is 50 to 60 mm and the mean compression rate per minute is 100 to 120. Any value outside these ranges was classified as “out of range.”

According to Bangor et al,^25^ a product with System Usability scores above 70 is considered acceptable, and ResuGlove’s System Usability score was calculated to be 70.4 of a maximum value of 100. The mean scores and SD of individual items were also calculated to look for the specific aspects of the System Usability Scale statements (Supplemental Digital Content, Supplementary Table 1, http://links.lww.com/JCN/A346). Statement 7, “I think that most people would learn to use this product quickly,” received the highest average rating with a mean of 4.41 (SD, 0.82), followed by statement 3, “I thought the product was easy to use,” reversed mean of 4.1 (SD, 0.62). Statement 8, “I find the product very uncomfortable to use,” received the third-highest score, with a reversed mean of 4.07 (SD, 1.19). In contrast, statement 4, “I would need technical support to learn how to use this product,” got the lowest average rating with a reversed mean of 3.03 (SD, 1.35) (Supplemental Digital Content, Supplementary Table 1, http://links.lww.com/JCN/A346).

## Discussion

In this study, we aimed to evaluate the effectiveness and usability of ResuGlove in improving the quality of CPR during simulated cardiac arrest scenarios in training sessions with manikin. Previous individual studies on the efficacy of AVF devices have yielded conflicting results for CPR training and resuscitation of actual patients.^17,33–37^ However, our finding is consistent with most systematic reviews of AVF devices, indicating improvement in at least 1 of the chest compression parameters.^10,12^ The ResuGlove significantly increased the percentages of adequate compression depth and chest recoil but did not influence the mean compression depth and rate. Similarly, compared with the standard CPR group, a higher percentage of participants in the ResuGlove CPR Group achieved adequate mean compression depth and rate. Our finding corresponds with a previous study that reported an increase in the percentage of participants who achieved the target compression rate and depth when an AVF device was used to guide rescuers' performance during CPR.^38^

Previous systematic reviews of similar studies suggest that, despite improvement in mean chest compression depth with AVF devices, it still does not meet the recommended value of international resuscitation guidelines.^10^ In contrast, this study found no significant difference in the mean compression depth between the groups. The mean compression depth was within the recommended range in both the ResuGloves CPR Group and the Standard CPR Group. Therefore, no improvement is expected in participants who performed CPR with the ResuGlove because the mean compression depth without the AVF device was already within the recommended range. The differences in the quality of chest compression depth across various studies could be due to variations in the stiffness or thickness of the chest wall of the manikins. Studies have found that manikins are not uniform in their compression characteristics,^39^ and the stiffness of the chest significantly influences the quality of chest compression depth.^40^ In addition, this study included young and healthy university students who had just completed basic CPR simulation training, which might have contributed to their better chest compression performance. Studies have shown that CPR skills tend to deteriorate over time unless rescuers undergo frequent training to maintain their knowledge and skills.^41^ Rescuers become less competent in CPR as time passes, and those who recently received training are better equipped to perform high-quality CPR.^42^

Consistent with a previous finding,^43^ the ResuGlove CPR Group had a significantly higher percentage of compressions with sufficient depth, although no improvement was noted in mean compression depth. Lakomek et al^43^ found that the mean depth of chest compressions did not improve with real-time AVF activation. However, there was a significant improvement in the percentage of adequate compression depth. Another study has shown that AVF devices can increase the percentage of rescuers who perform chest compressions within the recommended depth range.^38^ Our study also found this to be true: a significantly higher percentage of participants in the ResuGlove CPR Group achieved a mean compression depth within the recommended range of 50 to 60 mm.

In many studies, chest compression rates tend to decrease with the use of the AVF device compared with the standard CPR,^10,12^ although they remain within the recommended rate of 100 to 120.^10,12^ This study also shows a slight decrease in compression rates per minute in the ResuGlove CPR group (mean, 111.48; SD, 5.32) compared with the Standard CPR group (mean, 113.38; SD, 9.97) (Figure 3), although no significant difference was found between the groups. However, a significantly higher percentage of participants could perform compressions within the recommended mean compressions of 100 to 120 per minute, which aligns with previous findings.^38^ For example, in the ResuGlove CPR Group, only 1 participant (3%) had a compression rate greater than 120. In contrast, in the Standard CPR Group, 6 participants (20%) compressed the chest more than 120 times per minute. This finding is crucial because optimizing compression rates and depth is essential for better outcomes in CPR.^44^ In addition, faster compression rates are linked to reduced compression depth and overall compression quality.^45^

Maintaining complete chest recoil between each chest compression during CPR is critical, as leaning to the chest during chest compression can increase intrathoracic pressure, reducing perfusion pressure and cardiac output.^46^ As a result, complete chest recoil between each chest compression is essential for better outcomes after cardiac arrest. However, previous systematic reviews of standalone AVF devices did not demonstrate significant improvement in the quality of chest recoil using the AVF devices.^10^ Therefore, 1 of the goals of developing ResuGlove was to improve the detection capacity of learning, thereby ensuring full chest recoil between compressions.^23^ In the development process of the ResuGlove, pressure sensor input was used alongside acceleration measurements to accurately detect chest recoil.^23^

In our study, the Wilcoxon signed-rank test showed a significant difference favoring the ResuGlove CPR group in enhancing complete chest recoil during compressions. Whereas the median for adequate chest recoil remains unchanged, the mean values demonstrate a clear difference between the 2 groups (ResuGlove CPR Group vs Standard CPR Group, 93.1 vs 79.6). In addition, the lower quartile was higher in the ResuGlove CPR group (89.5) than in the Standard CPR group (53.5), reinforcing the superiority of ResuGlove in enhancing complete chest recoil (Figure 4). This is because the Wilcoxon signed-rank test concentrates on the distribution of paired observations rather than the central tendency potentially yielding significant *P* values even if the medians of the 2 groups are identical.^47^ The initial results of this study are promising, as the newly developed ResuGlove appears to be superior to standard CPR in detecting leaning and improving chest recoil.

The ResuGlove seems as effective as other standalone AVF devices in enhancing chest compression depth and rate. In addition, it appears to outperform other standalone AVF devices in detecting leaning and improving chest recoil. Furthermore, the ResuGlove is designed to be soft, flexible, and lightweight, making it comfortable to wear.^23^ This means that rescuers can wear them comfortably without experiencing discomfort, unlike rigid AVF devices, which can cause discomfort and might lead to low-quality compressions.^17,48^ In addition, the sensors are located on the rescuer’s palm, allowing direct and stable contact with the compression site, making it superior to other AVF devices, such as smartphones and smartwatches, in terms of minimizing errors.^17^ For example, in a study by Park,^17^ participants in the Standard CPR Group performed higher-quality compressions compared with participants in the Smartphone Feedback Group. The reason for the reduced performance when using the smartphone as feedback was the rescuers' hand pain and an unstable posture when handling the device.^17^

The System Usability score is interpreted in different ways, including determining usability through grading (A to F), descriptive adjectives for Usability (best imaginable to worst imaginable), and categorizing acceptability (acceptable to nonacceptable). We used the acceptability categorization method to interpret the usability score of this study. ResuGlove received a System Usability score of 70.4, which is acceptable but leans toward the lower end, suggesting that the device needs improvement in terms of usability. Our results are consistent with previous studies in which the usability of the newly developed wearable AVF devices to monitor chest compression quality was within an acceptable range.^21,49^

According to most previous evidence, the primary purpose of the System Usability Scale is to calculate a single reference score.^25^ In this study, however, individual statements' mean scores and SD were also calculated to examine each statement’s specific aspect. Item 7, concerning learnability, and item 3, regarding ease of use, received the highest average rating, with the lowest SD (Supplemental Digital Content, Supplementary Table 1, http://links.lww.com/JCN/A346). This indicates that most responses were clustered tightly around the mean, suggesting ResuGlove is not complex to learn and use. Ahn et al^21^ assessed the usability of a wearable smart ring AVF device that monitors the quality of chest compressions during CPR. In line with our findings, the researchers found the device to be user-friendly and easy to learn, as reflected in the participants' responses to the System Usability Scale questionnaire. Similarly, LaPrad et al^50^ used a tool other than System Usability Scale to assess the usability of a different wearable AVF device (smartwatch) and found that, in comparison with the defibrillator-based feedback, the smartwatch-based feedback device was generally perceived to be easier to operate and learn.

In contrast, item 4, which asked whether participants needed technical support to use the device, received the lowest mean and highest SD, indicating more significant variation in participants' responses. This suggests that even easy-to-learn and use devices may require some training before being used for the first time.

Research indicates that rigid and inflexible standalone AVF devices can cause discomfort for the rescuers' hands,^14–17^ potentially leading to quicker exhaustion during CPR. One of the goals of the textile-based flexible and soft wearable ResuGlove AVF device is to minimize discomfort to the rescuers' hands during CPR. Item 8, which inquiries about any discomfort participants may experience when using the device, received the third-highest score, with a reversed mean of 4.07 (SD, 1.19). This indicates that most participants in this study found the device comfortable. Consistent with our findings, a study conducted by Ahn et al^21^ reported that item 8, which assesses discomfort, received a high converted mean score of 3.65, suggesting that the smart ring AVF device was also comfortable to use during CPR. Similarly, LaPrad et al^50^ reported that a wearable smartwatch-based AVF device provided more comfort than a defibrillator-based AVF device. The findings from the usability assessment of this and prior studies indicate that wearable AVF devices may be ideal for alleviating the discomfort reported with rigid, hand-held, or chest-mounted AVF devices used during CPR.

It is possible that several factors in this study influenced some of the participants to give lower ratings to the System Usability Scale statements, causing the System Usability score to fall into the lower portion of the acceptable zone. For example, the ResuGlove prototype was explicitly designed for rescuers who prefer to place their right hand at the bottom during chest compressions. Consequently, all participants in the study used the same technique, despite some having indicated a preference for placing their left hand at the bottom. This might have negatively influenced the usability rating of those who are uncomfortable putting their right hand at the bottom during chest compressions. In addition, it was observed that, despite the compression being corrected, the device continued to provide corrective audio feedback for a few seconds until it could detect that the error had been corrected. This may be frustrating for participants, resulting in a lower usability rating. Moreover, the ResuGlove used in this study was only available in a single size. Therefore, some rescuers may experience discomfort because of the device being too tight and causing sweating, which could impact the System Usability score.

Some limitations should be considered when reading and interpreting the study’s findings. The ResuGlove is equipped with an audio feedback system that prioritizes feedback based on the preset criteria because, so far, it cannot simultaneously detect and provide multiple compression errors. For instance, in this study, compression depth was prioritized, as it is the most common chest compression parameter performed incorrectly, according to previous studies.^10^ This means that the device initially checked the quality of the compression depth and provided feedback if it was not performed correctly. Other parameters would not be corrected even if done incorrectly until the prioritized parameter is brought back into the recommended range.

The ResuGlove has only undergone testing on controlled simulated manikins, and participants were asked to perform chest compressions for only 2 minutes. Cardiopulmonary resuscitation in simulated cardiac arrest is not the same as CPR in real-life situations, where a patient’s life depends on the rescuer’s actions, and rescuers are usually in a highly stressful situation. Therefore, the high-quality chest compression reported in this study might not be accurate during actual patient resuscitation.

The study may not represent a real-world CPR situation in terms of the demographic characteristics of the participants. The study had a gender imbalance, with predominantly female participants who were all young and healthy. This differs from real-life CPR scenarios, where individuals of all ages, genders, educational backgrounds, and health conditions are expected to be involved. Moreover, although most bystanders in out-of-hospital cardiac arrest who attempt CPR usually have basic CPR training, pretty like our samples, there can be instances where those without prior training start CPR. Because ResuGlove is still in early development, future studies on ResuGlove will consider including individuals without any resuscitation experience.

## Conclusions

The study confirmed that the ResuGlove device, with its soft and flexible design, effectively guided the rescuers to deliver better-quality chest compressions during CPR. This is expected to reduce the discomfort experienced by rescuers when using rigid standalone AVF devices. In addition, the ResuGlove is a wearable device with sensors securely placed on the rescuer’s palm to minimize the risk of sensor displacement during compression, often seen in other standalone AVF devices. However, the device’s usability score was at the lower end of the acceptable range, indicating the need for further improvement. Finally, the positive outcome of this study is expected to stimulate further research and device development, leading to more cost-effective wearable AVF devices with additional features and better user interfaces to improve the quality of cardiovascular resuscitation.

## Acknowledgments

The authors are pleased to acknowledge Laerdal Medical, Vantaa, Finland, for lending us the Resusci Anne QCPR manikin with the QCPR app (iOS or Android), which was used in this study.
